# HLA Class I and Class II Alleles and Haplotypes Confirm the Berber Origin of the Present Day Tunisian Population

**DOI:** 10.1371/journal.pone.0136909

**Published:** 2015-08-28

**Authors:** Abdelhafidh Hajjej, Wassim Y. Almawi, Lasmar Hattab, Amel El-Gaaied, Slama Hmida

**Affiliations:** 1 Department of Immunogenetics, National Blood Transfusion Center, Tunis, Tunisia; 2 Department of Biochemistry, College of Medicine and Medical Sciences, Arabian Gulf University, Manama, Bahrain; 3 Department of Medical Analysis, Regional Hospital of Gabes, Gabes, Tunisia; 4 Laboratory of Immunogenetics, Department of Biology, University of Tunis – El-Manar, Tunis, Tunisia; Hospital Israelita Albert Einstein, BRAZIL

## Abstract

In view of its distinct geographical location and relatively small area, Tunisia witnessed the presence of many civilizations and ethnic groups throughout history, thereby questioning the origin of present-day Tunisian population. We investigated HLA class I and class II gene profiles in Tunisians, and compared this profile with those of Mediterranean and Sub-Sahara African populations. A total of 376 unrelated Tunisian individuals of both genders were genotyped for HLA class I (A, B) and class II (DRB1, DQB1), using reverse dot-blot hybridization (PCR-SSO) method. Statistical analysis was performed using Arlequin software. Phylogenetic trees were constructed by DISPAN software, and correspondence analysis was carried out by VISTA software. One hundred fifty-three HLA alleles were identified in the studied sample, which comprised 41, 50, 40 and 22 alleles at HLA-A,-B,-DRB1 and -DQB1 loci, respectively. The most frequent alleles were HLA-A*02:01 (16.76%), HLA-B*44:02/03 (17.82%), HLA-DRB1*07:01 (19.02%), and HLA-DQB1*03:01 (17.95%). Four-locus haplotype analysis identified HLA-A*02:01-B*50:01-DRB1*07:01-DQB1*02:02 (2.2%) as the common haplotype in Tunisians. Compared to other nearby populations, Tunisians appear to be genetically related to Western Mediterranean population, in particular North Africans and Berbers. In conclusion, HLA genotype results indicate that Tunisians are related to present-day North Africans, Berbers and to Iberians, but not to Eastern Arabs (Palestinians, Jordanians and Lebanese). This suggests that the genetic contribution of Arab invasion of 7^th^-11^th^ century A.D. had little impact of the North African gene pool.

## Introduction

HLA molecules are divided into class I and class II molecules, and are encoded by genes found on the short arm of chromosome 6 (6p21.3), and is one of the most polymorphic regions of the human genome [1]. The high diversity in HLA loci resides in exons 2 and 3 for class I genes, and in exon 2 for class II genes, and their selective ethnic distribution is the result of functional polymorphisms [2]. Analysis of the gene flow between different populations may be measured from the corresponding genetic distances from HLA allele frequencies, and thus HLA genotype analysis has proved to be useful in defining the origin of specific ethnic groups. DNA typing techniques has facilitated identification of a large number of HLA alleles, which generally correlate with geographic location of populations, hence confirming the utility of HLA genotyping in population studies [3, 4]. A striking feature of HLA alleles is the strong linkage disequilibrium (LD) between alleles at different HLA loci, in which HLA alleles may be associated in populations more frequently than expected based on their gene frequencies. This indicates an evolutionary relationship between specific HLA alleles.

Tunisians are the descendants of indigenous Berbers, and of people from civilizations which invaded or migrated to Tunisia throughout history. The latter include Phoenicians (ancestors of present-day Lebanese), who settled in Tunisia (Carthage) in the 8^th^ century B.C. By this time, there were 100,000 Phoenicians and 500,000 Berbers in Tunisia plus another 2.5 million Berbers in the rest of North Africa. The Roman rule era followed, and extended until the 5^th^ century, and was succeeded by the invasion of the European tribes, including Vandals [5]. A significant admixture of the Tunisian population was with the Islamic invasion of North Africa in 7^th^ century A.D. by Arabian Peninsula and Middle Eastern population [6,7], which was followed by the Egyptian (Fatimids) invasion in 11^th^ century A.D. [8]. More recently, Tunisia was subjected to the Turkish (Ottoman) rule which extended over 400 years, which was accompanied with the European conquest and migration of Africa and the Middle East in the 19^th^ century [9]. Tunisia eventually became a French protectorate, until the formal independence from France in 1956.

Geographically, Tunisia is the smallest (area: 164,000 km^2^) of the Maghreb (North African) countries. Current Tunisian population is estimated at 11 million [10], with high ethnical diversity, which comprised Berbers who live in isolated communities in Matmata region, and speak both Shleuh (*chleuch*; Berber language) and Arabic, along with Negroid (black) and Arabic-speaking groups. Here we investigated the genetic relatedness between Tunisian and North African, as well as other Mediterranean populations, using detailed characterization of HLA class I and class II loci analysis. This will improve our understanding of the origin and diversity of present-day Tunisian people.

## Material and Methods

### Study subjects

Three hundred and seventy-six Tunisian individuals of both genders were recruited into the study. All individuals included in the present study were unrelated, without any sign of clinically diagnosed diseases, and randomly selected from different regions of Tunisia (North, South and Center). Informed and written consent to participate in the study was obtained from all study subjects; consent being approved by participating institutions. Research & ethics committees of National Blood Transfusion Center (Tunis, Tunisia) and University of Tunis–El-Manar (Tunis, Tunisia) approved the protocol of the study, which was according to the Helsinki declaration. The recruited individuals were subjected to HLA class I (A, B) and class II (DRB1, DQB1) high-resolution genotyping and phylogenetic calculations. Genomic DNA was prepared from peripheral mononuclear cells using salting-out method [11]. DNA purity and concentration were assessed by measuring the absorbance at 260 nm and 280 nm. The makeup of the other populations included for comparative purposes are detailed in Table 1.

**Table 1:** Population used for the present work.

Identification	Region and population	n	References
1	Tunisians	376	Present study
2	Gabesians	95	[38]
3	Palestinians	165	[30]
4	Berbers	140	[34]
5	Jordanians	146	[29]
6	Northern Tunisians-A	104	[33]
7	Libyans	118	[28]
8	Northern Tunisians-B	101	[13]
9	Moroccans-Agadir	98	[36]
10	Algiers	102	[27]
11	Spanish	176	[42]
12	Moroccans	98	[24]
13	Basques-Arratia	83	[47]
14	French	179	[43]
15	Italians	284	[43]
16	Ashkenazi-Jews	132	[44]
17	Basques	82	[42]
18	Sardinians (Sar)	91	[43]
19	Ghannouchians	82	[35]
20	Moroccans-Jews	94	[44]
21	Cretans	135	[41]
22	Macedonians	172	[39]
23	Lebanese-KZ	93	[26]
24	Lebanese-Yohmer	81	[26]
25	Lebanese-NS	59	[26]
26	Oromo	83	[26]
27	Greeks-Cyprus	101	[26]
28	Greeks	85	[26]
29	Rimaibe	39	[26]
30	Mossi	42	[26]
31	Portuguese	66	[26]
32	Turks	228	[45]
33	Turks-A	250	[37]
34	Amhara	98	[26]
35	Egyptians	101	[26]
36	Japanese	495	[43]
37	French-Rennes	200	[13]
38	Greeks-A(Attica/Aegean)	85	[26]
39	Bubi	101	[26]
40	Catalans	88	[25]
41	Senegalese	103	[26]
42	Madenka	200	[26]
43	Bushmen	66	[43]
44	Tunisians-C	100	[31]

n: number of individuals analyzed for each population.

### HLA DNA genotyping

High-resolution HLA class I (A, B) and class II (DRB1, DQB1) genotyping were performed by reverse dot blot hybridization (PCR-SSO) using high-resolution commercial kits (Innogenetics, ‘fujirebio- Europe’, N.V. Zwijndrecht, Belgium) [12, 13]. This consisted of amplifying DNA using 5’-biotin labeled primers, followed by hybridizing PCR products with oligonucleotide probes immobilized on membrane-based strips. Biotinylated hybrid was detected by using streptavidin-labeled with alkaline phosphatase, followed by the addition of BCIP/NBT chromogen. In case of ambiguities or suspected homozygosy, the results were confirmed with PCR-SSP high-resolution kits manufactured by One-lambda (kitttridge street, Canoga Park, CA, USA)

### Statistical Analysis

HLA class I (A, B) and class II (DRB1, DQB1) allele frequencies were calculated by simple gene counting. Haplotypes frequencies were estimated by maximum-likelihood (ML) from genotypic data using expectation-maximization (EM) algorithm [14, 15], using the Arlequin program v2.0.1 [16]. Hardy-Weinberg equilibrium (HWE) was tested by applying Markov chain, as modified by Guo and Thompson with 100,000 iterations [17]. Linkage disequilibrium (LD), defined as the non-random association of 2 loci on the same chromosome, and the level of significance (*P*) for 2 × 2 comparisons and the relative linkage disequilibrium (RLD; D’) were calculated as previously described [18]. Phylogenetic trees (dendrograms) were constructed from individual allele frequencies by the Neighbour-Joining (NJ) method [19], with standard genetic distances (SGD) [20], using DISPAN software [21, 22]. Three-dimensional correspondence analysis, and bi-dimensional representation, was carried out using VISTAV5.02 software [23]. Correspondence analysis comprises a geometric technique, used for displaying a global view of the relationship among populations according to HLA (or other) allele frequencies, was based on allele frequency variance among populations, and on the display of a statistical projection of the differences.

## Results

### HLA allele frequencies in the studied population

The distributions of HLA class I and class II genotypes were in HWE in the studied Tunisian population. Table 2 presents the frequencies of HLA-A, -B, -DRB1 and -DQB1 alleles in Tunisians. In total, 153 HLA alleles were identified in the studied sample. Of the forty-one HLA-A alleles identified in the tested Berber sample, A*02:01, commonly observed in Moroccan Berbers (17.8%) [24] and Basques (27%) [25], was the most frequent allele in Tunisians (16.76%). The next frequent HLA-A allele seen in Tunisians was A*34:02 (9.31%), which is also present in high frequencies in North African and Mediterranean populations [26]. For HLA-B, fifty alleles were detected (Table 2). B*44:02/03 (17.82%) and B*51:01 (6.78%) alleles were the most frequent in Tunisian studied population. B*44 is a common allele in several common Mediterranean and Arab-speaking populations [27–31], and high frequencies of B*44 were reported in Swiss (27.7%) [26], and in French (23.2%) and Spanish (21.7%) Basques [32]. On the other hand, B*51:01 was commonly reported in other Tunisian and Arab-speaking populations [24, 27, 33]. Forty HLA-DRB1 alleles were detected; the most frequent allele is DRB1*07:01 (19.02%), which is present at high frequencies in Tunisian Berbers (17.6%) [34] and Ghannouch (28.7%) [35]. A high frequency of DRB1*03:01 (15.6%) allele was observed in Tunisia, which was comparable to frequencies established earlier for Moroccans (17.3%) [36], Tunisians (15.1%) [33], Algerians, and some European populations [37] including Spanish Basques (19.3%) [25]. At the DQB1*locus, 21 alleles were detected, of which DQB1*03:01 was the most frequent (18.0%), followed by DQB1*02:01(16.8%) and DQB1*02:02 (15.2%), which were also reported for Tunisians elsewhere [33–35, 38]

**Table 2:** HLA-A, - B, -DRB1 and -DQB1 Allele Frequencies (2n: 752).

Locus	Allele	Frequency	Locus	Allele	Frequency
HLA-A*	01:01	0.0798	HLA-B*	50:04	0.0173
	02:01	0.1676		51:01	0.0678
	02:02	0.0013		51:04	0.0013
	02:04/05	0.0332		51:08	0.0066
	02:11	0.0013		51:09	0.0027
	02:16	0.0027		52: 01:01	0.0199
	02:20	0.0013		53:01	0.0106
	02:27	0.0013		55:01	0.0146
	02:29	0.0013		56:01	0.0040
	02:36	0.0013		57:03	0.0093
	03:01	0.0492		58:01	0.0199
	03:02	0.0013		58:02	0.0013
	11:01	0.0226		82:01	0.0027
	11:04	0.0040		82:02	0.0013
	23:01	0.0678	HLA-DRB1*	01:01	0.0066
	24:02	0.0718		01:02	0.0519
	24:07	0.0027		01:03	0.0013
	24:10	0.0013		03:01	0.1556
	24:13	0.0080		03:02	0.0093
	24:14	0.0013		03:03	0.0013
	24:16	0.0080		03:07	0.0013
	25:01	0.0013		03:08	0.0013
	26:01	0.0146		03:09	0.0013
	26:13	0.0013		03:21	0.0013
	29:01	0.0532		04:01	0.0093
	30:01	0.0319		04:02	0.0372
	30:02	0.0346		04:03	0.0558
	30:03	0.0013		04:04	0.0093
	30:04	0.0173		04:05	0.0199
	30:07	0.0013		04:06	0.0119
	31:01	0.0040		04:07	0.0013
	32:01	0.0532		07:01	0.1902
	32:02	0.0146		08:01	0.0093
	33:01	0.0346		08:02	0.0080
	34:02	0.0931		08: 03	0.0027
	36:01	0.0106		08:04	0.0093
	66:01	0.0146		09:01	0.0066
	68:02	0.0585		10:01	0.0332
	68:06	0.0013		11:01/04	0.0864
	69:01	0.0199		11:02	0.0186
	74:01	0.0093		11:03	0.0093
HLA-B*	07:02	0.0439		12:01	0.0040
	07:05	0.0040		13:01	0.0519
	08:01	0.0665		13:02	0.0279
	13:01	0.0013		13:03	0.0346
	13:02	0.0066		13:05	0.0013
	14:01	0.0053		13:34	0.0013
	14:02	0.0279		13:37	0.0013
	15:03	0.0199		14:01	0.0053
	15:10	0.0053		14:04	0.0027
	15:16	0.0093		15:01	0.0811
	15:17	0.0093		15:02	0.0160
	18:01	0.0319		15:03	0.0106
	18:02	0.0013		16:01	0.0080
	27:02	0.0173		16:02	0.0040
	35:01	0.0253	HLA-DQB1*	02:01	0.1676
	35:03	0.0040		02:02	0.1516
	35:05	0.0053		02:03	0.0080
	35:08	0.0120		03:01	0.1795
	35:32	0.0053		03:02	0.1104
	37:01	0.0013		03:03	0.0293
	38:01	0.0106		03:04	0.0027
	39:01:01	0.0173		03:05	0.0013
	39:04	0.0186		03:12	0.0053
	39:06	0.0053		04:02	0.0372
	40:02	0.0505		05:01	0.0997
	41:01	0.0253		05:02	0.0186
	41:02	0.0346		05:03	0.0066
	41:03	0.0027		06:01	0.0146
	42:01	0.0040		06:02	0.0971
	42:02	0.0013		06:03	0.0346
	44:02/03	0.1782		06:04	0.0239
	45:01	0.0306		06:06	0.0027
	47:03	0.0027		06:08	0.0013
	49:01	0.0253		06: 09	0.0053
	50:01	0.0838		06:14	0.0013
	50:02	0.0266		06:17	0.0013

### Allelic comparison between Tunisians and other populations

**Fig 1:**
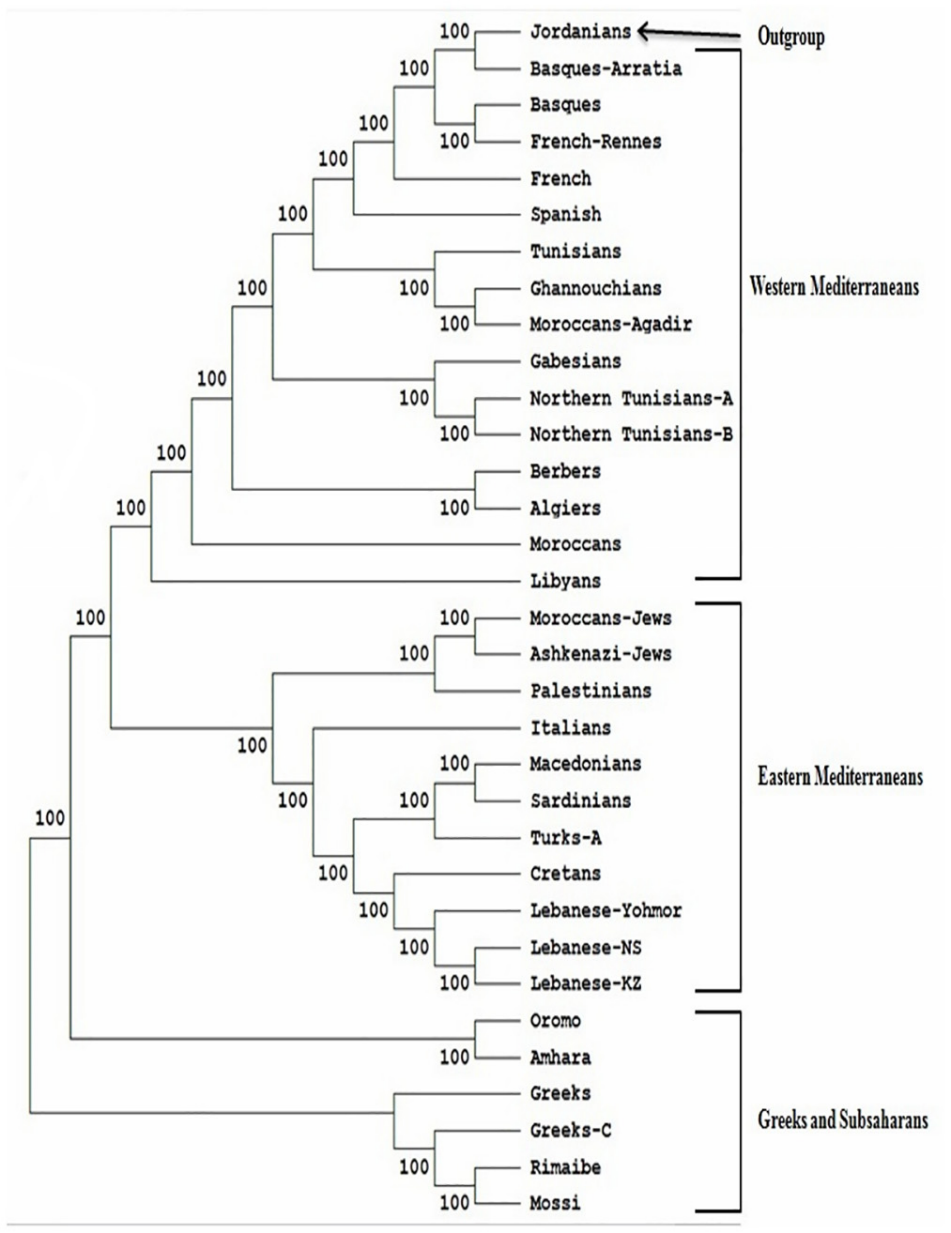
Neighbor-Joining dendrogram showing relatedness between Tunisians and other populations. Standard genetic distances (SGD) between populations were calculated by using high resolution HLA-DRB1 genotyping. Data from other populations were taken from references detailed in Table 1. Bootstrap values from 1.000 replicates are shown. Only Individuals with defined DRB1 subtypes are considered.

**Fig 2:**
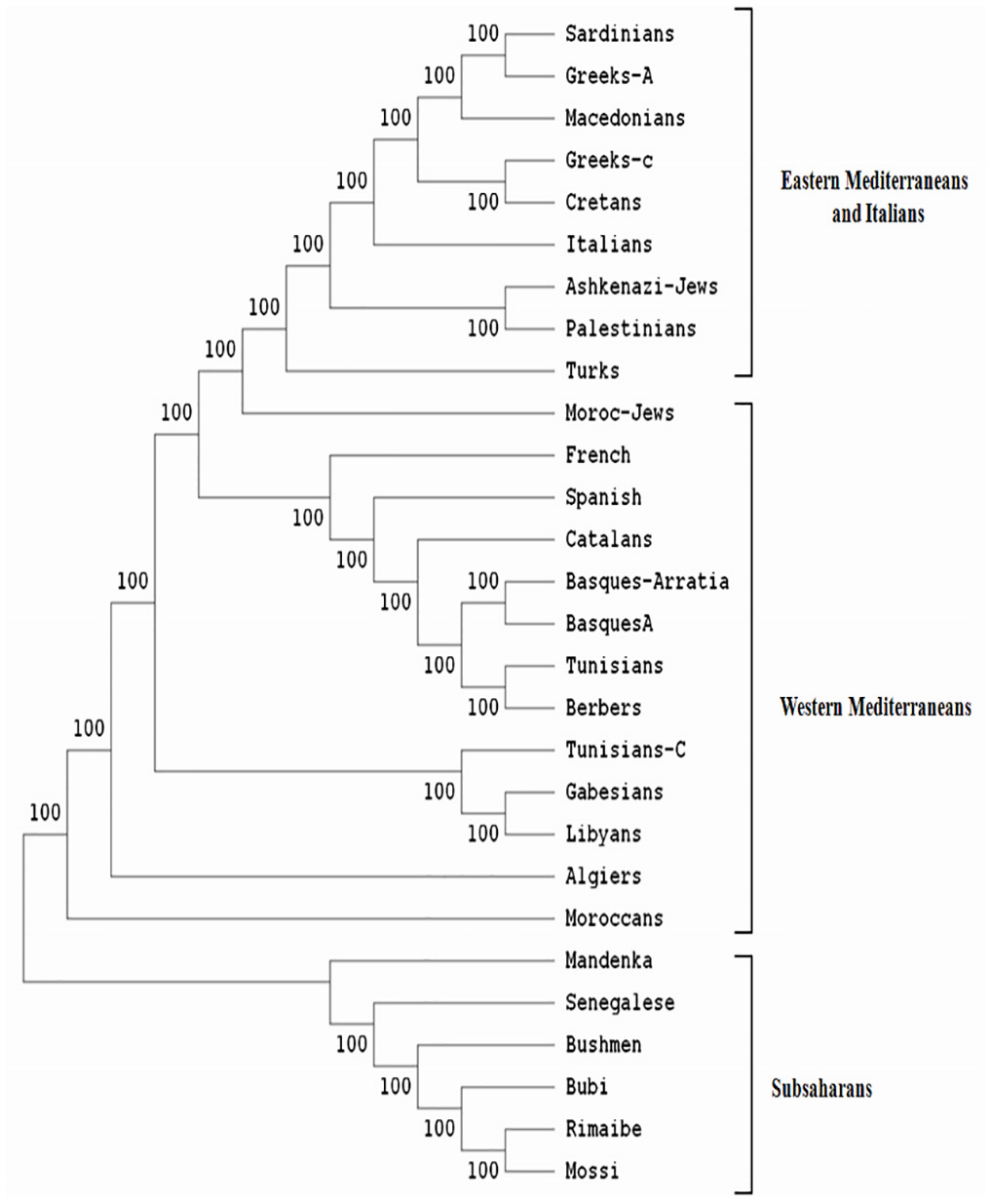
Neighbor-Joining dendrogram showing relatedness between Tunisians and other populations. Standard genetic distances (SGD) between populations were calculated by using generic HLA-B genotyping. Data from other populations were taken from references detailed in Table 1. Bootstrap values from 1.000 replicates are shown.

**Fig 3:**
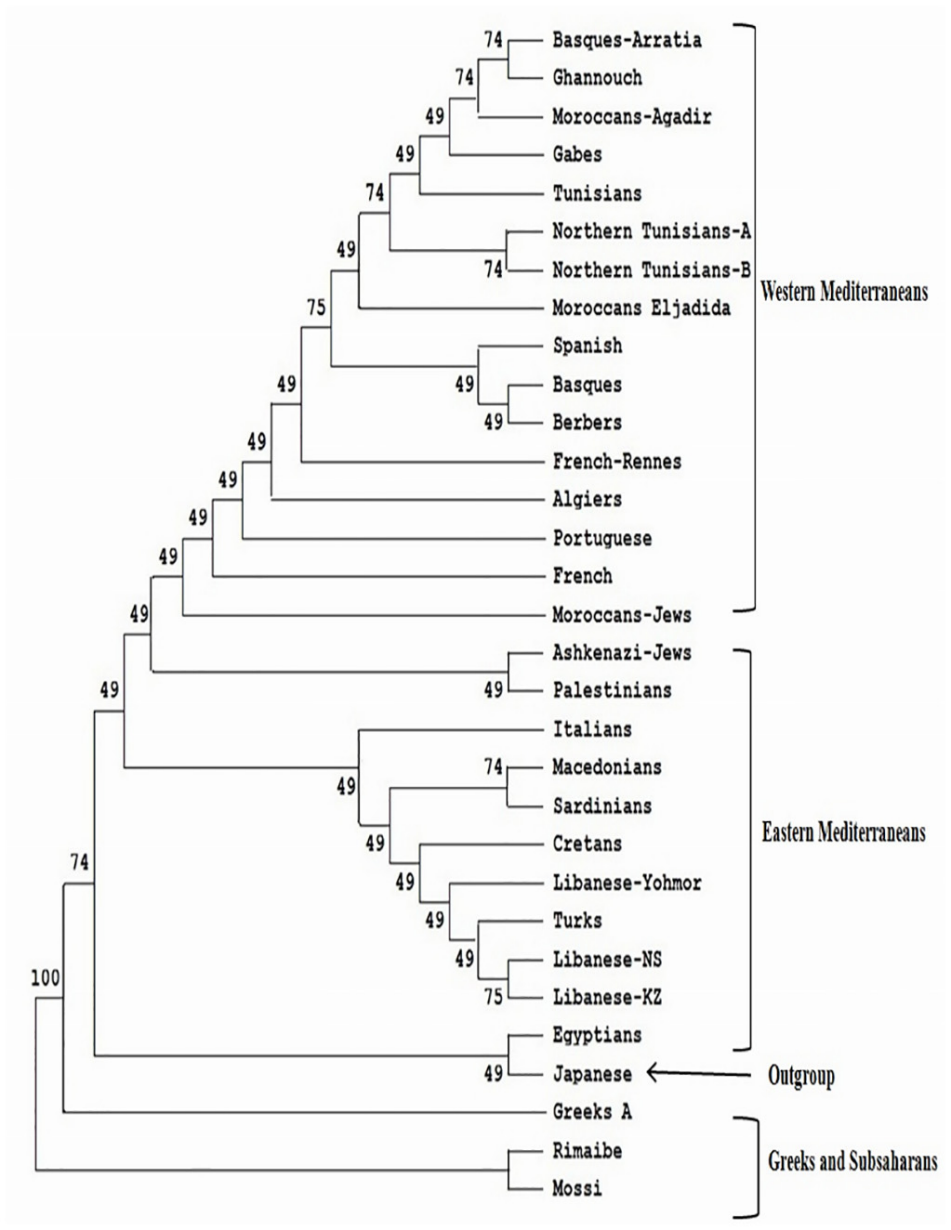
Neighbor-Joining dendrogram showing relatedness between Tunisians and other populations. Standard genetic distances (SGD) between populations were calculated by using generic HLA-DRB1 and -DQB1 genotyping. Data from other populations were taken from references detailed in Table 1. Bootstrap values from 1.000 replicates are shown.

**Fig 4:**
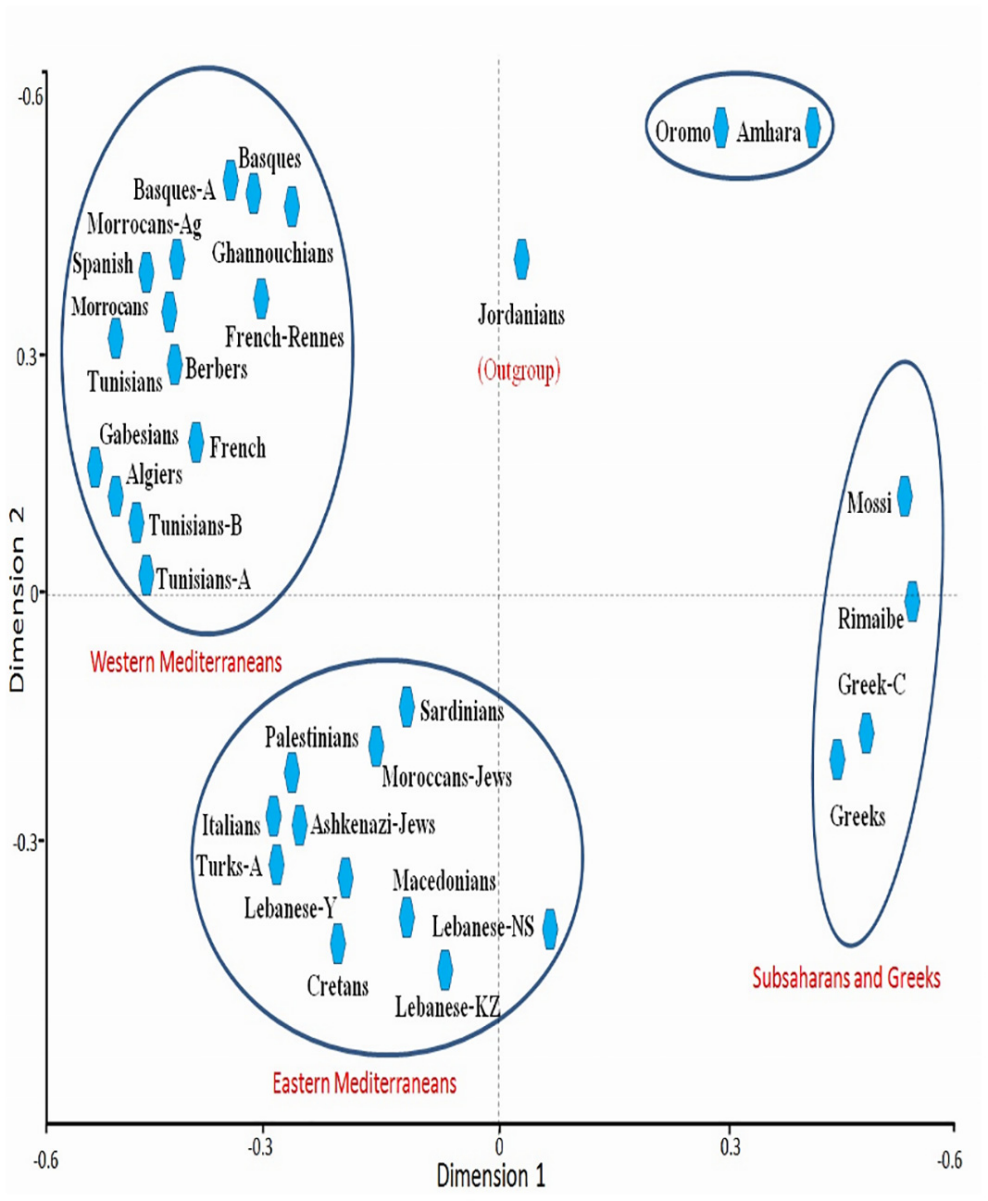
Correspondence analysis showing a global view of the relationship among Mediterranean populations according to HLA allele frequencies in three dimensions (bi-dimensional representation). HLA-DRB1 allele frequencies data. Only individuals with defined DRB1 subtypes are considered.

Some of the populations used for comparisons lacked HLA-A and B data [Lebanese (NS, kZ, andY), Moroccans-Agadir, Jews (Ashkenazi, Moroccans), French-Rennes, Tunisians], generic DQ[Greeks and Greeks-Cyprus], or high-resolution HLA-DRB1 data [Catalans, Portuguese, Turks]. These partially HLA typed populations should have been ignored, but they could be analysed conjointly taking in account only either DRB1 or generic A, B, DR and DQ frequencies. In addition, the typing techniques and their resolution level are too different which can lead to a high disparity in data presentation and then comparison difficulties. The latter can sometimes affect the calculations of genetic distances and eventually, even slightly, comparisons between populations. These reasons explain the choice of populations, the HLA locus and the resolution level of typing data in each figure (Figs 1–4)

The comparison of Tunisian HLA frequencies to other Mediterranean population frequencies was carried out by three types of analysis: high resolution HLA-DRB1 data, which is probably more informative and discriminating methodology (Fig 1), with generic HLA- B data (Fig 2) and with generic HLA-DRB1 and DQB1 data (Fig 3). These types of analysis were performed to confirm our results, and as some of the populations included for comparison lacked high-resolution HLA-DRB1 data. The allelic comparison was done at Neighbor-Joining (Figs 1, 2 and 3), standard genetic distances (Table 3), and correspondence analysis (Fig 4).

**Table 3:** Standard genetic distances (SGD^1^) between Tunisians and other populations.

Population ^2^	SGD x 10^−2^	Population	SGD x 10^−2^
Gabesians	-1.2	Moroccans-Jews	17.38
Moroccans-Agadir	0.56	Turks-A	18.26
Berbers	0.80	Lebanese-Yohmor	20.21
Algiers	1.33	Cretans	23.95
Northern Tunisians-A	1.60	Oromo	34.36
Spanish	1.71	Sardinians	35.81
Northern Tunisians-B	2.86	Macedonians	36.07
Libyans	3.17	Lebanese-KZ	38.32
Moroccans	3.42	Jordanians	39.65
Basques-Arratia	4.72	Lebanese-NS	41.38
French	7.31	Amhara	48.88
Ghannouchians	8.86	Greeks	74.25
Basques	11.66	Greeks-Cyprus	90.62
French-Rennes	14.19	Bushmen	106.60
Italians	14.41	Mossi	151.29
Palestinians	14.42	Rimaibe	154.16
Ashkenazi-Jews	14.99		

^1.^ Obtained by using HLA-DRB1 allele frequencies.

^2.^ Refer to Table 1 for profile of populations studied.

#### Neighbor-Joining Dendrogram

Neighbor-joining dendrogram, using the standards genetic distances (SGD) based on the high resolution HLA-DRB1 data (Fig 1), showed that there is a steady gradient of relatedness between Western and Eastern Mediterranean populations. The branches of Neighbor-Joining present high bootstrap values: the main one divided into two sub-branches, one clustering together Western Europeans (Basques, Spanish, French), and North Africans including Tunisians, and other groups Eastern Mediterraneans (Turks, Palestinians, Cretans, Lebanese, Macedonians), Italians, and Moroccan Jews. The other branch includes Greek and Sub-Saharan African populations. These data was confirmed by the other analysis (Figs 2 and 3), using generic HLA-DRB1 and DQB1 data, carried out in this study [33, 34, 39–41].

#### Standard genetic distances comparison

Comparison of standard genetic distances between Tunisians and Mediterraneans showed that Tunisians are closer to Western than to Eastern Mediterranean populations. This was evident in Table 3, where Tunisians-A-Gabesians had the closest genetic distance (-1.2 × 10^−2^) followed by Moroccans-Agadir (0.56 × 10^−2^), Tunisian Berbers (0.80 × 10^−2^) Algiers (1.33 × 10^−2^), Northern Tunisians-A (1.60 × 10^−2^), Spanish (1.71 × 10^−2^), Northern Tunisians-B (2.86 × 10^−2^), Libyans and Moroccans. In contrast Tunisians were distant to Eastern Mediterranean Arab-speaking populations (Palestinians, Lebanese and Jordanians).

#### Correspondence analysis

Three main clusters were identified by the correspondence analysis, using high resolution HLA-DRB1 data (Fig 4). This grouped together Western Europeans and North Africans (including Tunisians), placing together Eastern Mediterranean populations, and locating together Sub-Saharans and Greeks. Jordanians were considered outside this grouping scheme. Data from Fig 4 revealed that on the basis of the HLA-system, Tunisians are related to North African and Iberian, as well as other Western Mediterranean populations. These results obtained by correspondence analysis, correlated with results obtained by standard genetic distances and Neighbor-Joining trees.

### HLA-A, -B, -DRB1 and -DQB1 linkage disequilibrium in Tunisians

Table 4 shows the HLA- two-loci haplotypes with significant linkage disequilibrium in Tunisians. The investigation of Tunisians HLA haplotypes permits their comparisons with those previously reported in other populations. Indeed, our data depict that the most common haplotypes in Tunisians were also observed in other Mediterranean populations. In addition, the most frequent two-locus HLA haplotypes seen were, particularly, DRB1-DQB1 haplotypes [DRB1*03:01-DQB1*02:01 (16.60%), DRB1*07:01-DQB1*02:02 (14.80%) and DRB1*15:01-DQB1*06:02 (07.80%)] because of the strong disequilibrium between HLA Class II alleles. These haplotypes were common in Mediterranean populations. Indeed, the DRB1*03:01-DQB1*02:01 haplotype was found in Northern Tunisians (14.08%) [33], Ghannouchians (17.66%) [35], Gabesians (18.42%) [38], Tunisian Berbers (11.26%) [34], Algerians (11.3%) [27], Basques (17.5%) [25], Moroccans (17.3%) [24] and Cretans (7.4%) [41]. Besides, DRB1*07:01-DQB1*02:02 was also present in Ghannouchians (16.46%) [35], Northern Tunisians (9.71%) [33], Tunisian Berbers (16.03%) [34], Spaniards (17.3%) [42] and Moroccans (12.6%) [24]. Except for the three haplotypes formerly mentioned, no high frequency HLA haplotypes are found in Tunisia. This may be due to the existence of a higher admixture of Mediterranean populations in this population. Therefore, the HLA-A-B haplotypes found in Tunisia reflect common characteristics with the other Mediterranean background (Table 4). These results confirm those obtained by allele frequency analysis.

**Table 4:** HLA Class I and Class II two-Locus haplotypes in Tunisians.

HLA	Haplotype	Frequency	D’	HLA	Haplotype	Frequency	D’
A-B	01:01–49:01	0.0150	0.23	DRB1-DQB1	01:01–05:01	0.0060	0.64
	01:01–57:03	0.0060	0.34		01:02–05:01	0.0240	0.64
	02: 01–44:02/03	0.0386	0.17		03:01–02:01	0.1660	0.86
	02:01–49:01	0.0142	0.29		03:02–04:02	0.0080	0.38
	02:01–50:01	0.0330	0.40		03:03–04:02	0.0020	1.00
	03:02–50:01	0.0040	0.46		04:02–03:02	0.0260	0.66
	23:01–50:02	0.0163	0.39		04:02–03:05	0.0020	1.00
	23:01–58:01	0.0060	0.18		04:03–03:02	0.0440	0.64
	24:02–07:05	0.0060	0.72		04:03–04:02	0.0120	0.26
	24:02–15:17	0.0040	0.45		04:04–03:02	0.0060	0.54
	24:02–39:06	0.0040	0.45		04:05–03:02	0.0240	0.65
	24:02–52:01:01	0.0136	0.41		04:06–03:03	0.0040	0.22
	24:13–14:03	0.0020	1.00		07:01–02:02	0.1480	0.88
	26:01–38:01	0.0057	0.49		07:01–02:03	0.0100	0.79
	29:01–45:01	0.0179	0.32		07:01–03:03	0.0280	0.63
	30:01–13:02	0.0040	0.58		08:01–04:02	0.0040	1.00
	30:02–08:01	0.0060	0.20		08:04–03:01	0.0060	0.70
	30:02–27:02	0.0060	0.20		10:01–05:01	0.0380	0.81
	30:07–18:01	0.0020	1.00		10:01–06:06	0.0020	1.00
	32: 01–40: 02	0.0080	0.24		11:01/04-03:01	0.0720	0.62
	32: 01–41:01	0.0180	0.55		11:02–03:01	0.0180	1.00
	32:01–41:03	0.0040	0.65		13:01–06:03	0.0240	0.84
	32:02–40:02	0.0060	0.41		13:01–06:09	0.0060	0.58
	33:01–14:02	0.0040	0.41		13:01–06:17	0.0020	1.00
	34:02–44:02/03	0.0182	0.17		13:02–06:04	0.0180	0.66
	34:02–08:01	0.0212	0.26		13:02–06:14	0.0020	1.00
	34:02–35:01	0.0100	0.38		13:03–03:01	0.0340	0.73
	36:01–58:01	0.0040	0.38		14:04–05:03	0.0040	1.00
	68:02–15:03	0.0060	0.25		15:01–06:02	0.0780	0.89
	69:01–44:02/03	0.0040	0.54		15:02–06:01	0.0200	0.81
	74:01–51:08	0.0040	0.66		16:01–05:02	0.0120	0.66

### HLA Class I and Class II Extended Haplotype Analysis

HLA-A-B-DRB1-DQB1 extended haplotype (and their frequencies) detected in Tunisians are shown in Table 5. The most frequent haplotype found in Tunisian population, A*02:01–B*50:01–DRB1*07:01–DQB1*02:02 (2.2%), was also found in Tunisian Berbers (8.1%) [34], Gabesians (2.6%) [38], Northern Tunisians (1.2%) [33], Ghannouchians (1.8%) [35], Spaniards (1.2%) [42], Italians (0.5%), Turks (1.3%) and Moroccan Jews (2%) [43–45]. In addition, A*24:02-B*08:01-DRB1*03:01-DQB1*02:01 haplotype (1.6%) is found in Gabesians (1.6%) [38], Ghannouchians (4.2%) [35], and frequently associated with A*1 in Southern Tunisians [46], Mediterraneans and in Spaniards (3.4%), Basques (5%), Macedonians (4.9%), Yugoslavians (7.7%) [18, 39, 43, 47]. A*34:02–B*44–DRB1*15:01–DQB1*06:02 haplotype (4.25%) [34], which characterizes Tunisian Berbers, was detected in the present Tunisian population at lower frequency (0.8%). Other HLA-A-B-DRB1-DQB1 extended haplotypes found in the Tunisian population indicate a Mediterranean background. These haplotype results are concordant with those obtained by allele frequency analysis (genetic distance, Neighbor-Joining trees and correspondence analysis).

**Table 5:** Most frequent HLA four- loci haplotypes in Tunisians.

A-B-DRB1-DQB1	Frequency
01:01–45:01–07:01–02:02	0.006
01:01–49:01–04:05–03:02	0.006
02:01–44:02/03-03:01–02:01	0.008
02:01–44:02/03–04:02–03:02	0.006
02:01–44:02/03-07:01–02:02	0.012
02:01–49:01–13:03–03:01	0.006
02:01–50:01–07:01–02:02	0.022
02:01–51:01–03:01–02:01	0.014
23:01–08:01–03:01–02:01	0.006
23:01–45:01–04:03–03:02	0.006
23:01–50:02–07:01–03:03	0.010
23:01–58:01–11:01/04-03:01	0.006
24:02–08:01–03:01–02:01	0.016
24:02–52:01:01–15:02–06:01	0.012
29:01–39:011–07:01–02:02	0.016
29:01–45:01–03:01–02:01	0.008
32:01–41:01–10:01–05:01	0.014
34:02–08:01–03:01–02:01	0.016
34:02–35:01–13:01–06:09	0.006
34:02–44:02/03–07:01–02:02	0.008
34:02–44:02/03-15:01–06:02	0.008
66:01–35:08–04:03–03:02	0.010
68:02–39:04–11:01/04-03:01	0.006

## Discussion

This study is the first to examine high resolution HLA class I (A, B) and class II (DRB1, DQB1) genotypes in a large sample of Tunisians comprising 376 individuals. HLA alleles data were used for calculating standard genetic distances, neighbor-joining dendrograms, correspondence analysis (CA), and the generation of extended HLA haplotypes. Frequent alleles and haplotypes found in the Tunisian population were also seen in Western Mediterranean populations, with similar frequencies. Genetic distance, Neighbor-Joining trees and correspondence analysis confirmed this relatedness, and correlates with those obtained by earlier studies in Tunisians [33, 34, 38–40].

### Tunisians and North Africans

The relatedness of Tunisians to North Africans is expected, given that North Africans share, albeit with minor differences, similar history. North Africa was originally populated by Berbers, who were successively invaded, first by the Phoenicians (1000 BC), and later by Greek invasions (457–404 BC), Roman Punic wars (264–266 BC), and Romans settlement in North Africa [48]. Later significant admixture of North Africans (including Tunisians) was brought about by the Muslim conquest of North Africa in the 7^th^ century AD, and the massive Bedouin immigration in 11^th^ century, followed by northern (Andalusians) and southern (Negroid slaves) migration [48, 49]. This points to the interrelatedness of North African populations.

### Tunisians and Eastern Arabs

Throughout history, Tunisia was subjected to a wave of Arabian invasions, which originated from the Arabian Peninsula [48]. The first invasion commenced in 647 AD, which included recruits from Medina (Saudi Arabia) and Memphis (Egypt), which targeted the Byzantine Exarchate of Africa [50]. Eventually, the Byzantine Empire was defeated in Africa, which was abandoned to Islamic empire [51]. The second wave of Arabian invasion came in the 11th century AD, which included Bedouin tribal recruits from Hijaz and Nejd regions of present day Saudi Arabia [52].

Current HLA study, which was based on Neighbor-Joining trees, correspondence analysis, genetic distances, and haplotype studies, suggests that Tunisians are distinct from other Middle Eastern Arab population (Palestinians, Lebanese and Jordanians), despite the Arab successive incursions that occurred in Tunisia. This indicates that the 7^th^ and 11^th^ centuries AD Arab invasion of North Africa did not affect the North African genetic pool; rather the Berber genetic profile was retained. It is possible that the number of newcomers from the Middle East was probably very low when compared to the existing Berbers. This was supported by the findings that the number of the invading Arabs did not exceed 40,000 thousands, and that the military campaign did not exceed eighteen months, after which most of the invading troops returned to the originating area [53]. By comparison, However, the number of newcomers in 11^th^ century was considerable higher (250,000) [54], an indication that the 7th century AD invasion was not followed by establishment of settlements.

The low contribution of the Arab influence into Tunisian genetic pool is explained by the absence of admixture between Berbers and Arabian tribes. It is noteworthy that most Berbers were forced to live in the mountains for fear of persecution during that time, which was enforced by cultural barriers (language, religion, traditions) between Berbers and Arabs. As such, the Arab influx was a major factor in the linguistic, cultural and ethnic Arabization of Tunisia, and in the spread of Islam and nomadism in areas where agriculture had previously been dominant [55]. While North Africans probably are genetically Berbers, but culturally Arabs.

### Tunisians and Iberians

In contrast to Arab comparative analysis, Neighbour-Joining trees, correspondence analysis, genetic distances, and haplotype studies showed that present-day Tunisians are more related to Iberians (Basques and Spaniards), in agreement with results published elsewhere [24, 38, 41, 47]. Additionally, Basque and Spaniard Iberians are closely related to North African Berbers and other present-day populations [33, 34]. This was supported by the fact that the genetic distances between Iberians and North Africans (including Tunisians) are markedly large with Eastern Mediterraneans and Arabs from Middle East (Palestinians, Lebanese and Jordanians).

This relatedness between Iberians and North Africans can be attributed to the similar history between Iberians and North Africans, as both were invaded by Phoenicians, Romans, Germans (Visigoths in Iberia, Vandals in North Africa), Muslim Arabs and Berbers [56]. The Islamic landing at Gibraltar in the 8th Century, and subsequent Islamic occupation of the Iberian Peninsula for 450 years [57], did not markedly alter the Iberian genetic pool, which retained its Berber substratum. This may be attributed to the fact that the number of Muslim invaders was relatively low (30,000) compared to native Iberians at the time (8 million) [34], and that most of the invaders were North African Berber recruits, who are genetically closer to Spaniards than to Arabs. In addition, cultural barriers minimized the admixture between Iberians and Muslims. As such, the relatedness between Iberians and North Africans (including Tunisians) can be attributed to the northward Saharan migration, which occurred in 10,000–4,000 BC, when the Berbers relocated to the Northern Mediterranean coast during hyper-arid conditions [41]. Accordingly, while the 7^th^-8^th^ –11th centuries AD Arab invasion of the area had low gene flow, it had strong social and cultural effects in both Iberia and North Africa.

In conclusion, our analysis, based on genetic Neighbour-Joining trees, correspondence analysis, genetic distances and haplotype construction, shows that the Tunisians are related to North Africans and Iberians (Basques and Spaniards), and that all these populations show big distances to Eastern Mediterraneans and Middle Eastern Arabs. Thus, present-day Tunisians are not genetically distinguishable from Tunisian Berbers and North Africa Berber populations, in spite of cultural differences (language) between them.
